# The Story of the Insane from Year to Year

**Published:** 1898-03-05

**Authors:** 


### THE STORY OF THE INSANE FROM YEAR TO YEAR.

Tenth Series.

### THE LANCASHIRE COUNTY ASYLUM AT RAINHILL.

An increase from 1,818 to 1,838 has to be noted. There were 321 first admissions, 18 readmissions, 125 recoveries, and 181 deaths. The recoveries stand at 36·87 per cent. of the admissions, and the deaths at 9·90 per cent. on the average number resident. Cerebral and spinal diseases account for 64 of the deaths, consumption for 42, and enteritis for 2. Of the year's admissions not fewer than 76 were driven insane by drink and 67 by hereditary influence. High as these numbers are there is no doubt they would be higher still if the whole truth could be arrived at; and nevertheless drinking and marriage among tainted people go on just as if no penalty were likely to follow them. Additional accommodation to the extent of 20 beds was opened during the year.

